# Time and visual-spatial illusions: Evidence for cross-dimensional interference between duration and illusory size

**DOI:** 10.3758/s13414-023-02737-x

**Published:** 2023-06-29

**Authors:** Daniel Bratzke, Lena Peris, Rolf Ulrich

**Affiliations:** 1 Department of Psychology, University of Bremen, Bremen, Germany; 2 Department of Psychology, Eberhard Karls University of Tübingen, Tübingen, Germany

**Keywords:** Time perception, Ebbinghaus illusion, Horizontal-vertical illusion, Space–time interaction

## Abstract

Time and space are intimately related to each other. Previous evidence has shown that stimulus size can affect perceived duration even when size differences are illusory. In the present study, we investigated the effect of visual-spatial illusions on duration judgments in a temporal reproduction paradigm. Specifically, we induced the Ebbinghaus illusion (Exp. 1) and the horizontal-vertical illusion (Exp. 2) during the encoding phase of the target interval or the reproduction phase. The results showed (a) that illusory size affects temporal processing similarly to the way physical size does, (b) that the effect is independent of whether the illusion appeared during encoding or reproduction, and (c) that the interference between size and temporal processing is bidirectional. These results suggest a rather late locus of size-time interference in the processing stream.

## Introduction

Time, space, and quantity are probably tightly linked in our minds (Walsh, 2003), and it is often assumed that time and space share a common spatial representation (Cai & Connell, 2015; Casasanto & Boroditsky, 2008; Eikmeier et al., 2013; Winter et al., 2015). The link between time and space has been investigated at a conceptual level (e.g., Boroditsky, 2000; Ulrich et al., 2012) and at a fundamental perceptual level (e.g., Casasanto & Boroditsky, 2008). There is now ample evidence that spatial stimulus attributes shape the perception of time, such as physical size (e.g., Mo & Michalski, 1972; Rammsayer & Verner, 2014; Thomas & Cantor, 1975; Xuan et al., 2007), imagined stimulus size (e.g., Birngruber & Ulrich, 2019), and movement (e.g., Brown, 1995). Some theorists have argued that time and space can be subsumed within a common magnitude system (Walsh, 2003), which serves as a universal mental metric for a variety of quantitative dimensions, including numerical magnitude, luminance, size, color saturation, and also time (Alards-Tomalin et al., 2014; Xuan et al., 2007).

To investigate the locus of the size effect on time perception within the processing stream, Ono and Kawahara (2007) measured the perceived duration of a visual object (an empty circle) and manipulated its subjective size via the Ebbinghaus illusion. In this illusion, a central circle is perceived as smaller when surrounded by large compared to small inducer circles. Participants had to indicate the perceived duration after each presentation of the illusion by pressing one of four keys (from “1” for short to “4” for long). In two experiments, Ono and Kawahara observed that the Ebbinghaus illusion influenced the perceived duration of the central circle in the same way as one would expect with physical size. More precisely, subjectively larger stimuli were perceived as longer than subjectively smaller stimuli. This size-duration effect was observed irrespective of whether size judgments were assessed within the same trial as duration judgments (Exp. 1) or in different blocks (Exp. 2). In Experiment 3, the authors ruled out that the effect was due to a stronger competition for attentional resources by the larger surrounding inducer circles. Based on the assumption that the Ebbinghaus illusion arises at a relatively late processing stage, the authors concluded that visual processing exerts its influence on temporal processing at a relatively late stage.

Effects of non-temporal attributes on temporal processing have often been interpreted within the theoretical framework of internal clock models (e.g., Creelman, 1962; Gibbon et al., 1984; Treisman, 1963; Wearden, 2016; Zakay & Block, 1997). These models assume that a pacemaker elicits pulses that are accumulated by a counter as long as a switch is closed. The number of accumulated pulses represents time and can be compared with other representations from reference memory. Effects of non-temporal stimulus attributes on perceived duration can arise directly at the clock mechanism –at the switch, pacemaker, or memory/decision level. Importantly, many magnitude effects on time perception show the signature of pacemaker effects; that is, they increase with increasing stimulus duration (for an overview, see Fig. 3 in Matthews & Meck, 2016; Wearden, 2016). Accordingly, a larger magnitude should be associated with a higher pacemaker rate (a “switch effect” due to different detection times for the stimulus on- and offsets should be constant across stimulus durations).

Several studies have used the reproduction method to investigate size effects on time perception. This method is usually considered less susceptible to decision biases than comparison methods (e.g., Matthews & Meck, 2016; Rammsayer & Verner, 2014). With this method, the manipulation of interest can be applied during the encoding of the target interval or during the reproduction phase. What is important here is that internal clock models predict opposite effects for these two conditions if the effect is located at the clock mechanism. In the encoding condition, more pulses are collected during the target interval when the pacemaker runs faster, which leads to longer reproductions in the subsequent reproduction phase. In contrast, in the reproduction condition, a faster pacemaker rate during the reproduction phase leads to shorter reproductions because the desired number of pulses is reached sooner. These predictions have been met in several studies, for example, when comparing filled with unfilled intervals (Bratzke et al., 2017), auditory with visual stimuli (Bratzke & Ulrich, 2019), and flickering with static stimuli (Cai & Connell, 2016).

However, previous results with magnitude manipulations are somewhat inconsistent regarding the above prediction. For example, while Chang et al. (2011) observed the predicted pattern with numerical magnitude (1, 2 vs. 8, 9), a subsequent study failed to replicate this finding (Cai & Wang, 2014). More specifically, in the study by Cai and Wang the numerical size effect on temporal reproduction was replicated when the numerical stimulus appeared during the encoding of the target interval but not when it appeared during reproduction. This was even the case when participants had to report the numerical size of the stimulus after each trial (Exp. 3). Similar results were obtained by Rammsayer and Verner (2015) for physical stimulus size. Moreover, Cai and Connell (2016) observed that spatial distance (i.e., spatial distance between two lines) affected reproductions at encoding but not at reproduction, while visual flicker showed opposite effects between encoding and reproduction (longer reproductions for flickering than static stimuli at encoding vs. longer reproductions for static than flickering stimuli at reproduction). The lack of an effect on temporal reproductions when magnitude varies during reproduction has been regarded as evidence for a memory locus of the effect instead of emerging from the clock mechanism. For example, Cai and colleagues suggested that both non-temporal magnitude information and perceived duration are represented as mental magnitudes and, therefore, can interfere with each other when they are concurrently held in memory (Cai & Connell, 2016; Cai & Wang, 2014).

**Fig. 1:**
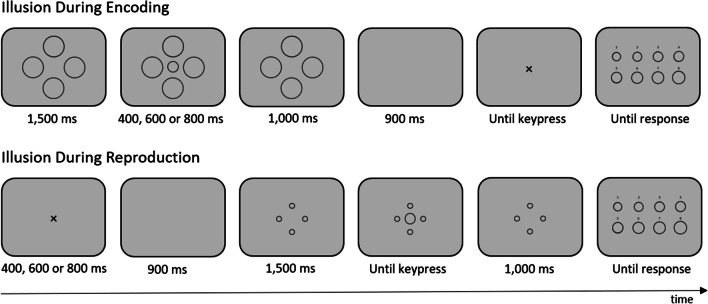
Trial structure in the encoding and the reproduction group in Experiment 1

In the present study, we aimed to replicate the effect of illusory size on time perception observed by Ono and Kawahara (2007) using the method of temporal reproduction. To localize the effect within the internal clock framework, we applied the illusion during the encoding of the target interval and during the reproduction (see Fig. 1 for an illustration of the two conditions). If the size-duration effect is located at the clock component, one would expect opposite effects depending on whether the illusion is induced during the encoding or during the reproduction phase. In contrast, if the effect is located at the memory component, one would expect a size-duration effect only when the illusion is induced during the encoding of the target interval. Furthermore, we aimed to extend the present approach to another visual illusion, the horizontal-vertical illusion. According to Coren et al.’s (1976) taxonomy of visual illusions, this illusion belongs to a different global class of visual illusions (illusions of extent) than the Ebbinghaus illusion (illusions of shape). Moreover, it comes with the advantage that no surrounding objects are necessary to induce the illusion.

## Experiment 1

In Experiment 1, illusory size was manipulated via the Ebbinghaus illusion. The procedure was very similar to Ono and Kawahara (2007), except that we used the temporal reproduction method and the illusion was applied during the encoding of the target interval and during the reproduction phase. In all trials, participants also had to estimate the size of the inner circle after the duration reproduction (as in Exp. 1 of Ono & Kawahara, 2007).

## Method

### Participants

One hundred and one volunteers participated for course credit. A power analysis (with the software G*Power 3.1; Faul et al., 2007) for a within-between interaction (power = .8, alpha = .05) yielded a required sample size of *N* = 200 for a small effect size (*f* = .01) and *N* = 34 for a medium effect size (*f* = 0.25). We opted for an intermediate sample size of *N* =100. Twenty-six participants had to be excluded as outliers (see *Data analysis*). Thus, the final sample consisted of 75 participants (39 in the encoding and 36 in the reproduction condition) with a mean age of 23.0 years (*SD* = 4.8; 51 female, 23 male; one participant did not report age and sex). All participants reported normal or corrected-to-normal vision and provided informed consent before data collection.

### Apparatus and stimuli

The experiment was an online experiment and run on the participants’ individual computers. It was created in PsychoPy (Peirce et al., 2019) and hosted by Pavlovia (https://pavlovia.org). The PsychoPy code *ScreenScale* (Morys-Carter, 2021) was used to adjust the screen scale on individual computers. The following visual angles refer to a viewing distance of 60 cm. The Ebbinghaus stimuli consisted of four outer inducer circles and one central circle. As in Ono and Kawahara (2007), the diameter of the inducer circles was 4° in the “subjective large” condition and 1° in the “subjective small” condition. The central circle always had a diameter of 2°. The distance between the inducer circles and the central circle was 1°. There were eight comparison circles, which ranged from 1.65° to 2.35°. Comparison circles were presented in ascending order from left to right in two rows of four circles each (see Fig. 1).

### Tasks and procedure

In the encoding condition (see upper part of Fig. 1), each trial started with presenting the four inducer circles for 1,500 ms. Then, the central target circle appeared for 400, 600, or 800 ms, together with the inducer circles. After offsetting the central circle, the inducer circles remained on the screen for another 1,000 ms. After an interstimulus interval of 900 ms, a cross (“ × ”) appeared on the screen, indicating the onset of the reproduction interval. Participants had to terminate the reproduction interval by pressing the spacebar. Finally, the array of comparison circles appeared, and participants had to indicate which of the eight circles resembled the central circle in the trial by typing in the corresponding number. The next trial started after a variable intertrial interval of 1,000 or 1,400 ms.

In the reproduction condition (see lower part of Fig. 1), each trial started with the presentation of the “ × ” for 400, 600, or 800 ms. After an interstimulus interval of 900 ms, the inducer circles were presented for 1,500 ms. Then, the central circle appeared, indicating the onset of the reproduction interval. The central circle disappeared when participants pressed the spacebar to terminate this interval. As in the encoding condition, the inducer circles remained on the screen for a further 1,000 ms. If participants pressed the spacebar before the presentation of the central circle, a message appeared on the screen for 1,000 ms, reminding them to only attend to the central circle for reproduction. The remainder of the trial was identical to the encoding condition.

There was a practice block of 12 trials, and five experimental blocks of 24 trials each (a total of 120 experimental trials). Thus, each combination of the two subjective size conditions (small vs. large) and the three target durations (400, 600, and 800 ms) was repeated 20 times. There were two groups of participants, one for the encoding and one for the reproduction condition.

### Data analysis

The outlier procedure was similar to the one applied by Chang et al. (2011). Accordingly, trials with reproductions shorter than 100 ms and longer than 1,600 ms were discarded (3.9% of all trials). Next, we discarded trials with reproductions that deviated more than 2 *SD*s from the mean reproduction for each combination of participant, subjective size, and target duration (4.8% of remaining trials). In a further step, we calculated individual linear regressions between reproduction and target duration. Twenty-three participants with non-significant (*n* = 22) or significant negative slopes (*n* = 1) were excluded from further analyses.^1^ Results for the whole sample are provided in Appendix A (Table 1 and Fig. 6). Separate mixed ANOVAs with the between-subjects factor *manipulation phase* (encoding vs. reproduction) and the within-subjects factors *subjective size* (small vs. large) and *target duration* (400, 600, or 800 ms) were then conducted for mean reproduction and mean size estimates. Degrees of freedom were corrected using the Greenhouse–Geisser correction whenever appropriate.

## Results and discussion

### Reproduction

**Fig. 2:**
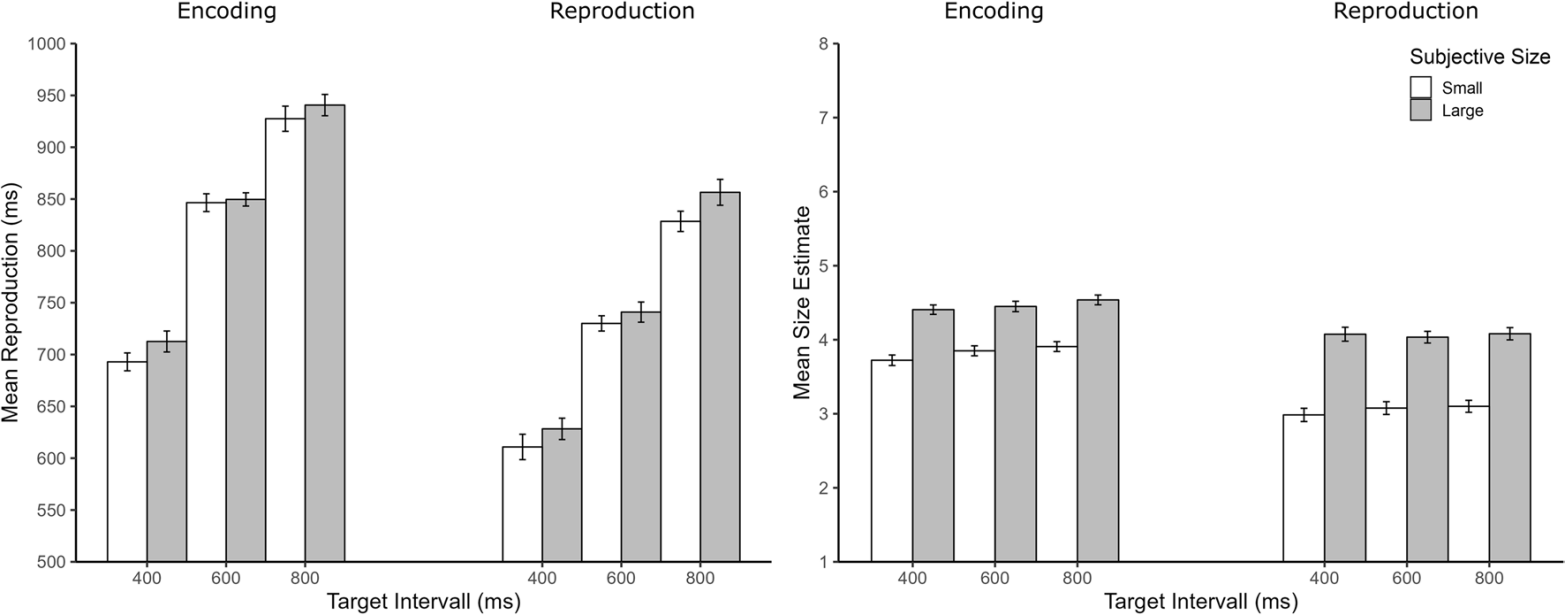
Mean reproduction (**left panel**) and mean size estimate (**right panel**) as a function of manipulation phase, target interval, and subjective size in Experiment 1. Error bars represent ± 1 within-subjects *SE* according to Morey (2008)

The results of Experiment 1 are depicted in Fig. 2. ANOVA for mean reproductions revealed significant main effects of manipulation phase, *F*(1, 73) = 9.05, *p* = 0.004, η_p_^2^ = 0.11, size, *F*(1, 73) = 11.67, *p* = 0.001, η_p_^2^ = 0.15, and target duration, *F*(2, 146) = 363.56, *p* < 0.001, η_p_^2^ = 0.85. Mean reproductions were longer when the Ebbinghaus illusion was applied during encoding (828 ms) than during reproduction (733 ms). Mean reproductions were also longer for the subjectively large (small inducers: 790 ms) than for the subjectively small (large inducers: 775 ms) size. Importantly, there were no significant two-way interactions, all *p*s ≥ 0.313*,* and no significant three-way interaction, *F*(2, 146) = 0.24, *p* = 0.784, η_p_^2^ < 0.01.

### Size estimates

As expected and consistent with the Ebbinghaus illusion, size estimates were affected by subjective size, *F*(1, 73) = 82.34, *p* < 0.001, η_p_^2^ = 0.53, with a mean size estimate of 4.30 for the subjectively large and 3.45 for the subjectively small condition. More interestingly, size estimates were also affected by manipulation phase, *F*(1, 73) = 7.84, *p* = 0.007, η_p_^2^ = 0.19, and target duration, *F*(2, 146) = 9.27, *p* < 0.001, η_p_^2^ = 0.11. Mean size estimates were larger when the Ebbinghaus illusion was applied during encoding (4.15) than during reproduction (3.59), and increased with increasing target duration (3.81, 3.86, and 3.92). The interaction between manipulation phase and subjective size was significant, *F*(1, 73) = 4.19, *p* = 0.044, η_p_^2^ = 0.05, as was the interaction between subjective size and target duration, *F*(1, 73) = 3.32, *p* = 0.039, η_p_^2^ = 0.04. The Ebbinghaus illusion (small inducers minus large inducers) was slightly larger in the reproduction (1.0) than in the encoding (0.6) condition, and the illusion decreased with increasing target duration (1.09, 0.98 and 0.96). The remaining two-way interaction and the three-way interaction were not significant (manipulation phase × target duration: *F*(1, 73) = 1.84, *p* = 0.162, η_p_^2^ = 0.02; three-way interaction:* F*(2, 146) = 0.24, *p* = 0.787, η_p_^2^ = 0.56).

Taken together, subjective size induced by the Ebbinghaus illusion affected temporal reproductions in the same way as one would expect with stimuli of different physical size; subjectively larger circles were reproduced longer than subjectively smaller circles. The results of Experiment 1 thus replicated the previous results by Ono and Kawahara (2007) with a different timing method. More importantly, however, subjective size influenced temporal reproductions irrespective of whether the Ebbinghaus illusion was induced during encoding or reproduction of the target interval. This result pattern is inconsistent with both of our hypotheses. It, however, resembles previous results with physical (Rammsayer & Verner, 2015) as well as symbolic size (i.e., numerical magnitude; Cai & Wang, 2014), with the difference that previous studies did not observe a size effect on reproductions when the manipulation was applied during the reproduction phase. One possible explanation for the size effect in the reproduction phase is that participants attended differently to the Ebbinghaus stimulus depending on the manipulation phase, namely to the target circle during the encoding and to the inducer circles (or the whole Ebbinghaus figure) during the reproduction. However, this explanation seems very unlikely because there was an even stronger Ebbinghaus illusion in the reproduction than in the encoding condition.

## Experiment 2

**Fig. 3:**
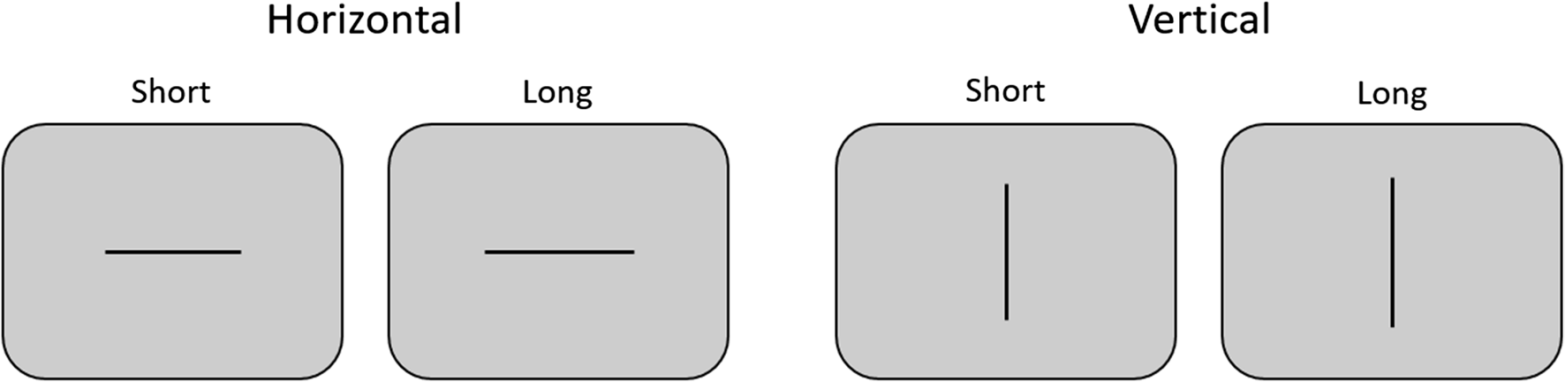
Stimuli for the horizontal-vertical illusion used in Experiment 2. For each variant, there was also a short and a long version (differences in line length are drawn to scale). Participants had to indicate whether the line was rather short or long

Experiment 2 was a conceptual replication of Experiment 1 using a variant of the horizontal-vertical illusion. In this illusion, vertical lines are perceived as being (spatially) longer than horizontal lines of the same length. In contrast to the Ebbinghaus illusion, no surrounding objects are necessary to induce the illusion, and thus any confounding between the physical size of the inducers and the subjective size of the target object is avoided. Instead of the classical T- or L-stimuli frequently used to induce the horizontal-vertical illusion (e.g., Avery & Day, 1969; Künnapas, 1955), we presented single lines in horizontal or vertical orientation. Moreover, to compare the effects of objective and illusory size on temporal processing more directly, we also varied physical line length (see Fig. 3).

## Method

### Participants

One hundred and fifty volunteers participated for course credit. One participant from the reproduction group was excluded because she had already participated in the encoding group. Another 67 participants had to be excluded as outliers; thus, the final sample consisted of 82 participants (49 in the encoding and 33 in the reproduction condition) with a mean age of 24.6 years (*SD* = 5.9; 68 female, 14 male). All participants reported normal or corrected-to-normal vision and provided informed consent prior to data collection.

### Apparatus and stimuli

The apparatus was the same as in Experiment 1. Stimuli were vertical and horizontal lines of two different lengths (9.5° and 10.5° of visual angle). They were presented at the center of the screen in black against a grey background.

### Tasks and procedure

The temporal course of a trial was similar to Experiment 1. In the encoding condition, each trial started with the presentation of one of the four lines for 400, 600, or 800 ms. After an interstimulus interval of 900 ms, the reproduction prompt (“ × ”) appeared and disappeared with the participant’s keypress. Participants were then asked to indicate whether the spatial extension of the line was rather short (with the “S” key) or rather long (with the “L” key), without providing them with an explicit reference. In the reproduction condition, each trial started with a presentation of the “ × ” for one of the three target durations. After 900 ms, one of the four lines appeared and indicated the start of the reproduction phase. After offsetting the line with the participant’s keypress, participants were asked to judge the spatial extension of the line. As in Experiment 1, the intertrial interval was either 1,000 or 1,400 ms (due to a programming oversight the encoding condition included an additional blank screen for 1,500 ms before each trial).

In the written instruction, it was emphasized that the judgment about the line length was only related to the spatial extension of the line. As in Experiment 1, the manipulation phase was a between-subject factor. In each experimental session, there was an initial practice block (24 trials) followed by five experimental blocks with 48 trials each (a total of 240 experimental trials). Thus, each combination of orientation (small vs. large), line length (short vs. long), and target duration (400, 600, and 800 ms) was repeated 20 times. Each experiment lasted about 45 min.

## Results and discussion

**Fig. 4:**
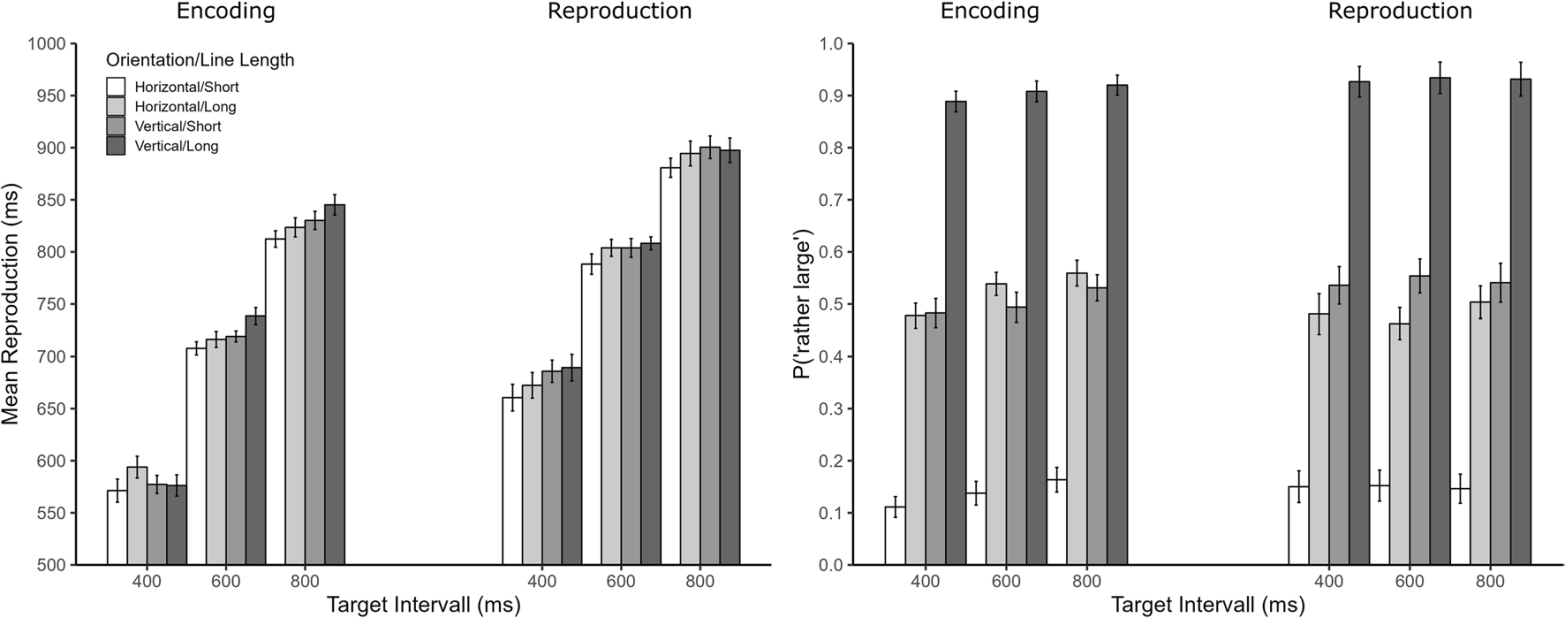
Mean reproduction (**left panel**) and relative frequency of “rather large” judgments (**right panel**) as a function of manipulation phase, target interval, line orientation and line length in Experiment 2. Error bars represent ± 1 within-subjects *SE* according to Morey (2008)

**Fig. 5:**
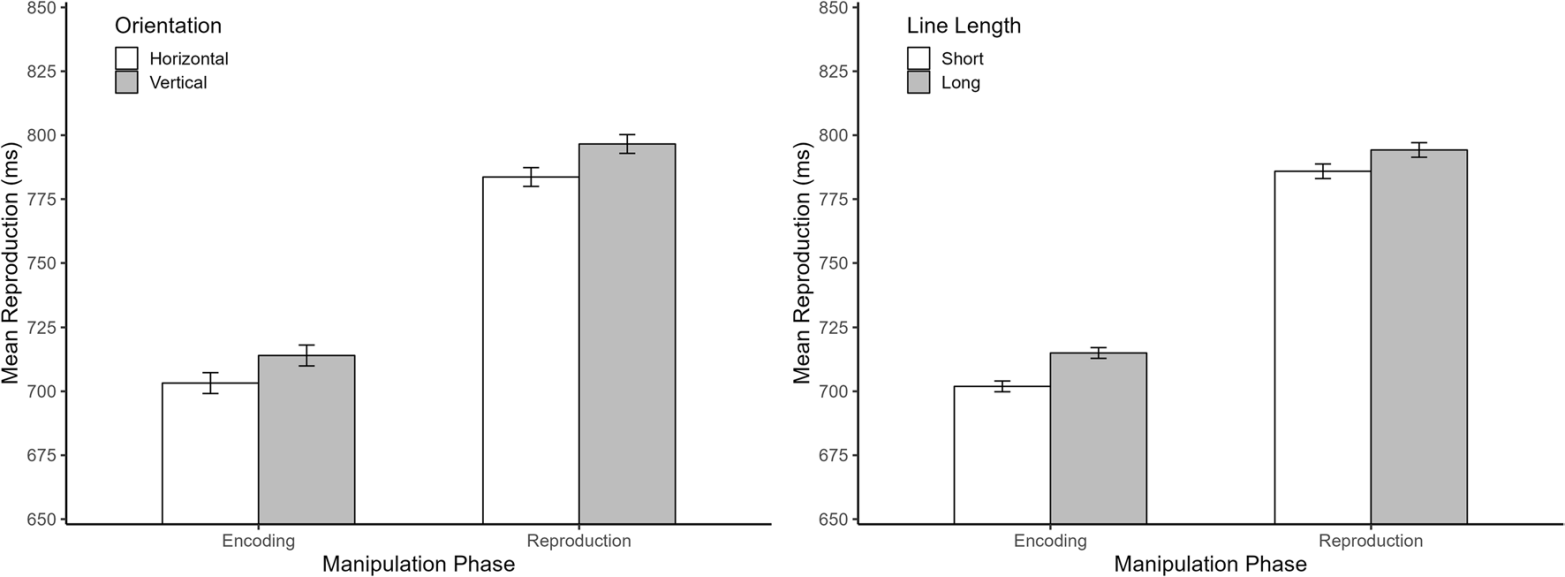
Mean reproduction as a function of manipulation phase and orientation (**left panel**) and as a function of manipulation phase and line length (**right panel**) in Experiment 2. Error bars represent ± 1 within-subjects *SE* according to Morey (2008)

**Fig. 6:**
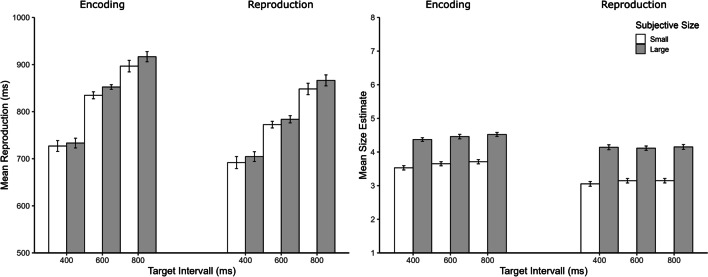
Mean reproduction (**left panel**) and mean size estimate (**right panel**) as a function of manipulation phase, target interval, and subjective size in Experiment 1 (including all participants). Error bars represent ± 1 within-subjects *SE* according to Morey (2008)

**Fig. 7:**
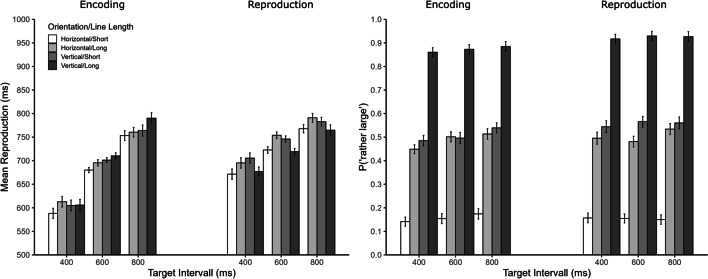
Mean reproduction (**left panel**) and relative frequency of “rather large” judgments (**right panel**) as a function of manipulation phase, target interval, line orientation and line length in Experiment 2 (including all participants). Error bars represent ± 1 within-subjects *SE* according to Morey (2008)

**Fig. 8:**
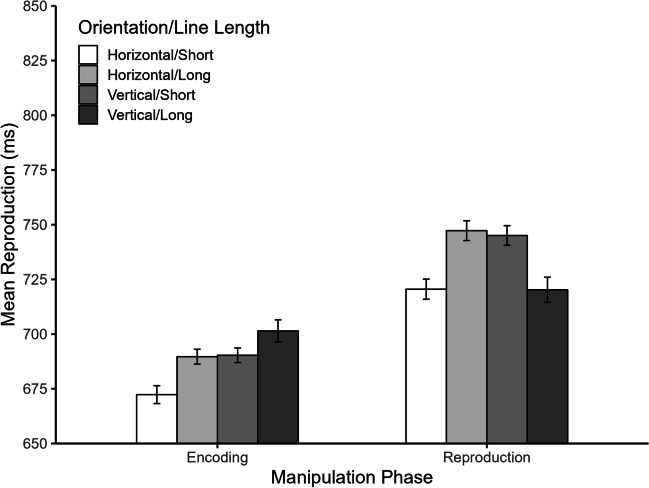
Mean reproduction as a function of manipulation phase, orientation and line length in Experiment 2 (including all participants). Error bars represent ± 1 within-subjects *SE* according to Morey (2008)

The outlier procedure was as in Experiment 1. Accordingly, trials with reproductions shorter than 100 ms and longer than 1,600 ms were discarded (6.7% of all trials), as were trials with reproductions deviating more than 2 *SD*s from the mean reproduction for each combination of participant, orientation, and target duration (4.6% of remaining trials). Sixty-seven participants with non-significant (*n* = 58) or significant negative slopes (*n* = 9) in the individual regression analyses were excluded from further analyses.^2^The results of Experiment 2 are depicted in Figs. 4 and 5. Results for the whole sample are provided in Appendix B (Table 2 and Figs. 7 and 8)

### Reproduction

ANOVA for mean reproductions revealed significant main effects of manipulation phase, *F*(1, 80) = 6.59, *p* = 0.012, η_p_^2^ = 0.08, orientation, *F*(1, 80) = 8.46, *p* = 0.005, η_p_^2^ = 0.10, line length, *F*(1, 80) = 20.94, *p* < 0.001, η_p_^2^ = 0.84, and target duration, *F*(2, 160) = 408.99, *p* < 0.001, η_p_^2^ = 0.84. Mean reproductions were longer in the reproduction (790 ms) than in the encoding group (709 ms), for vertical (748 ms) than for horizontal lines (736 ms), and also longer for long (747 ms) than for short (737 ms) lines. As expected, reproductions increased with increasing target duration (619, 753, and 854 ms). There was only one significant interaction, namely between manipulation phase, orientation, and target duration, *F*(2, 160) = 3.87, *p* = 0.026, η_p_^2^ = 0.05, all other *p*s ≥ 0.181. As can be seen in Fig. 4, there was a slightly inverse orientation effect for the 400-ms target interval. Separate ANOVAs confirmed this for the two groups, which showed a significant interaction between orientation and target duration for the encoding group, *F*(2, 96) = 5.01, *p* = 0.008, η_p_^2^ = 0.09, but not for the reproduction group, *F*(2, 64) = 0.55, *p* = 0.532, η_p_^2^ = 0.02. As can be seen in Fig. 5, the effects of orientation and line length on mean reproduction did not differ between the two manipulation phases.

### Size estimation

As expected, the probability of responding “rather long” was strongly affected by orientation, *F*(1, 80) = 336.84, *p* < 0.001, η_p_^2^ = 0.81, as well as by line length, *F*(1, 80) = 359.82, *p* < 0.001, η_p_^2^ = 0.82, with almost identical numerical effects for orientation (vertical: 0.72 vs. horizontal: 0.33) and line length (long: 0.71 vs. short: 0.33). In contrast to Experiment 1, size judgments were not affected by manipulation phase, *F*(1, 80) = 0.06, *p* = 0.815, η_p_^2^ < 0.01, however, the main effect of target duration was significant, *F*(2, 160) = 8.69, *p* < 0.001, η_p_^2^ = 0.10. This effect interacted with manipulation phase, *F*(2, 160) = 3.73, *p* = 0.031, η_p_^2^ = 0.04. Size judgments increased with target duration in the encoding group (0.49, 0.52, and 0.54; *p* < 0.001) but not in the reproduction group (0.52, 0.53, and 0.53; *p* = 0.851). All other interactions were not significant, all *p*s ≥ 0.162.

In summary, the results of Experiment 2 were very similar to those of Experiment 1. Again, illusory size affected temporal reproductions irrespective of whether the illusion appeared during the encoding or the reproduction phase. Moreover, there was again bidirectional interference between size and time. More precisely, size estimates were also influenced by the duration of the target interval, even though this influence was only observed for the encoding condition. In contrast to Experiment 1, reproductions were on average longer in the reproduction than in the encoding condition. This difference might be due to the characteristics of the stimuli used in the two experiments (e.g., presentation of inducers before and after target circle in Exp. 1). However, differences in the absolute level of reproductions should be interpreted with caution, as they reflect between-subjects variability, and previous studies have shown a variety of patterns between experiments even when the same stimuli were used during the encoding and the reproduction phase (e.g., Cai & Wang, 2014).

## General discussion

In the present study, we investigated the interplay between illusory size and temporal processing, using a temporal reproduction task that should allow us to distinguish between a locus of the interference effect at the clock or the memory component of an internal clock model. Employing two distinct visual size illusions, the Ebbinghaus (Exp. 1) and the horizontal-vertical (Exp. 2) illusion, and an additional manipulation of physical line length (Exp. 2), we were able to show that illusory size can affect time perception in a similar way as physical size, with larger stimuli resulting in longer temporal reproductions. The present results thus replicate and extend previous results regarding the effect of physical (e.g., Rammsayer & Verner, 2014; Thomas & Cantor, 1975; Xuan et al., 2007), numerical (e.g., Oliveri et al., 2008; Vicario, 2011), imagined (Birngruber & Ulrich, 2019), and illusory size (Ono & Kawahara, 2007) on time perception. Moreover, both experiments revealed the same effect of illusory size on temporal reproductions, irrespective of whether the illusion was induced during the encoding or the reproduction of the target interval. While this pattern is inconsistent with our original hypotheses, it renders an effect locus at the perceptual (clock component) level very unlikely.

Why did we observe the same effect during encoding and reproduction, while others observed opposing effects (Chang et al., 2011) or no effect during reproduction (Cai & Wang, 2014; Rammsayer & Verner, 2015)? A possible answer is that the assessment of stimulus size during reproduction directly interfered with the duration reproduction (Exp. 1: large stimulus = long reproduction; Exp. 2: long stimulus = long reproduction). This kind of interference seems plausible for two reasons. First, participants had to provide temporal reproductions and size estimates within the same trial; therefore, the size information could not be ignored (or not attended). Second, size information was immediately available to the system as soon as the reproduction stimulus appeared, and therefore interference could easily occur during the duration reproduction.

At first glance, several previous results seem to conflict with this explanation. First, Cai and Connell (2016) did not observe an effect of line length when it was varied during reproduction. In their study, however, participants did not have to reproduce or judge the line length. Additionally, since these authors used a continuous keypress for duration reproductions, their participants could have done the reproduction in principle without any attention to the line stimulus. Second, Cai et al. (2018) presented their participants with a constant-length line that consisted of two line segments of different lengths and colors, and a color cue indicated which segment had to be reproduced after the duration reproduction. They observed that line length affected reproductions only when the cue was provided before the duration information was retrieved from memory but not afterwards. In this case, however, it is possible that participants did not retrieve the length information until it was needed for the line reproduction, but instead just kept in mind the color information up to that point in time. In Rammsayer and Verner’s (2015) study, participants had to terminate the reproduction by pressing one of two keys depending on the stimulus size (or the shape in a control condition). Nonetheless, the authors did not observe a statistically significant size effect on reproductions; however, there was a positive numerical difference in temporal reproductions between physically large and small stimuli (presented at reproduction) for two of three target intervals (a mean difference of about 15 ms across the three target intervals with *p* = 0.22 and *N* = 30). Taken together, it still seems reasonable to us that the need to directly assess the spatial information during reproduction for a later judgment might be crucial for the effect of stimulus size on duration estimates to occur.

As mentioned before, cross-dimensional interference between space and time has been interpreted as resulting from memory interference when size and duration information are concurrently held in working memory (Cai & Connell, 2015; Cai & Wang, 2014; Cai et al., 2018). This interpretation was strongly motivated by the lack of cross-dimensional interference during the reproduction phase in previous studies. In a strict sense, such memory interference cannot explain our current observation of interference during the reproduction phase, as the duration information should already be retrieved from memory when the spatial information (illusory and physical size or line length) is presented as a reproduction signal. However, it still seems possible that both representations can interfere with each other even when the duration reproduction is already under way, as the size information is immediately available at the beginning of the reproduction phase. Alternatively, interference could arise at a later response level due to a decisional bias leaving both representations essentially unaffected (e.g., Yates et al., 2012). These alternative explanations can hardly be distinguished, yet a decisional bias seems less likely to us for two reasons. First, the method of reproduction has been regarded as the temporal judgment method that most directly assesses perceived duration and neither relies on category ratings nor utilizes comparative judgments, which both may be especially susceptible to decisional biases (cf., Rammsayer & Verner, 2014). Second, there is previous evidence that loading verbal working memory does not affect space–time interference, whereas loading visuospatial memory does, suggesting that online verbalization is not crucial for space–time interference (Starr & Brannon, 2016). All in all, we cannot decisively interpret our results in favor of a memory interference or a decisional bias explanation. It is even possible that the interference effects during encoding and reproduction reflect different kinds of interference, for example, memory interference in the former and a decisional bias in the latter case, or that both act simultaneously.

The present results showed bidirectional interference between temporal reproductions and size estimates. That is, not only size information affected duration judgments, but also stimulus duration affected size estimates, with larger size estimates for longer durations. This interference appeared mainly during the encoding phase (another hint that the interference mechanisms might differ between encoding and reproduction). Ono and Kawahara (2007) reported a similar effect in their first experiment (their second experiment showed a non-significant numerical difference in the same direction); however, most previous temporal reproduction studies using stimuli of varying physical size did not assess size estimates (Cai & Connell, 2016; Rammsayer &Verner, 2104, 2015, 2016). Two exceptions are the studies by Cai and Connell (2015) and Cai and Wang (2022), which also reported bidirectional interference and demonstrated that time-on-space effects are especially likely to occur when the spatial representation is rather noisy; for example, when it is conveyed by haptic stimulation (without visual feedback; Cai & Connell, 2015) or by dynamic stimulation (an unfilled line demarcated by two boundaries, which are presented asynchronously; Cai & Wang, 2022). Cai and colleagues (e.g., Cai & Connell, 2015) have regarded this bidirectional (or symmetric) cross-dimensional interference as evidence against a spatial metaphor account (which assumes that time processing is abstract but that spatial metaphors are employed when thinking or talking about time, e.g., Boroditsky, 2000) and in favor of the notion of a shared representational format for time and space. Recent results further suggest that cross-dimensional interference is not just a contextual effect but that for the interference to occur, the magnitude dimensions must belong to the same object (Togoli et al., 2022).

One could argue that in our Experiment 1, there was a confound between subjective (physical) size and numerical size; participants had to indicate the size of the stimuli by typing in a corresponding number, and thus physical and numerical size were explicitly associated. However, this was not the case in Experiment 2, where participants had to indicate whether the line appeared to them as rather short or rather long. This procedure, however, comes with the possibility of semantic overlap between the (explicit) size estimate and a memory tag representing the relative subjective duration of the target interval (e.g., “rather short” vs. “rather long”). In studies like the present one, such representational overlap can hardly be avoided because people usually talk about time in terms of space (e.g., Haspelmath, 1997). Explicit overlap could only be avoided by not asking participants for judgments of the respective stimulus attribute. However, this is only possible when a manipulation of stimulus attributes is salient (e.g., chromatic vs. achromatic stimuli) or intrusive (e.g., auditory pitch or loudness) so that one does not need to verify a difference in the percepts, or implicit measures are available for verification (e.g., brain activation, behavioral consequences).

Another potential limitation of the present study is the high outlier rate, which might indicate that the tasks and/or the online assessment posed some operational or motivational challenges to the participants, thereby compromising the validity of the data. However, to our knowledge, the remaining sample sizes still represent the largest ones to date with this particular paradigm (sample sizes in previous studies were often about half of the present ones). Together with the fact that we observed the same result pattern with two different visual illusions and one physical size manipulation across two different experiments, this makes us reasonably confident about our data quality.

In conclusion, the present study showed that subjective size as induced by visual size illusions can interfere with temporal processing in a similar way to that induced by physical size. Subjectively (as well as objectively) larger stimuli were perceived as being presented longer than subjectively smaller stimuli. This effect was observed for the Ebbinghaus as well as the horizontal-vertical illusion, and thus generalizes across different visual size illusions. The result that the same effect was observed irrespective of whether the illusion was induced during encoding of the target interval or during its reproduction was not expected, but nonetheless argues against a locus of the effect at the clock mechanism of an internal clock. It rather suggests that space–time interference arises at a later conceptual processing level, which is further supported by the current observation of bidirectional cross-dimensional interference between size and duration judgments.

## Appendix A

Results of Experiment 1 including the data of all participants (*N* = 101)

**Table 1:** ANOVA results in Experiment 1 (including all participants)

Source	*Df*	Reproduction			Size estimate		
		*F*	*p*	η_p_^2^	*F*	*p*	η_p_^2^
MP	1,99	2.37	0.127*	0.02	5.68	0.019	0.05
S	1,99	14.32	< 0.001	0.13	140.51	< 0.001	0.59
TI	2,198	158.13	< 0.001	0.61	12.61	< 0.001	0.11
MP × S	1,99	< 0.01	0.946	< 0.01	1.67	0.200*	0.02
MP × TI	2,198	1.55	0.218	0.02	3.47	0.033*	0.03
S × TI	2,198	0.38	0.684	< 0.01	2.07	0.128*	0.02
MP × S × TI	2,198	0.17	0.840	< 0.01	0.63	0.536	0.01

MP = manipulation phase, S = subjective size, TI = target interval

Asterisks indicate qualitatively different results from the main analyses

## Appendix B

Results of Experiment 2 including the data of all participants (*N* = 148).

One participant was excluded due to excessively long reproductions (*M* = 2,776 ms).

**Table 2:** ANOVA results in Experiment 2 (including all participants)

Source	*df*	Reproduction			Size estimate		
		*F*	*p*	η_p_^2^	*F*	*p*	η_p_^2^
MP	1,146	2.36	0.126*	0.02	1.08	0.301	0.01
O	1,146	3.43	0.066*	0.02	474.99	< 0.001	0.76
L	1,146	8.91	0.003	0.06	443.99	< 0.001	0.75
TI	2,292	104.13	< 0.001	0.42	12.82	< 0.001	0.08
MP × O	1,146	5.05	0.026*	0.03	1.51	0.220	0.01
MP × L	1,146	7.31	0.008*	0.05	0.09	0.767	< 0.01
MP × TI	2,292	9.32	0.002*	0.06	3.40	0.038	0.02
O × L	1,146	14.02	< 0.001*	0.09	1.45	0.231	0.01
O × TI	2,198	0.02	0.984	< 0.01	0.46	0.629	< 0.01
L × TI	2,292	0.30	0.742	< 0.01	0.52	0.595	< 0.01
MP × O × L	1,146	9.95	0.002*	0.06	0.14	0.705	< 0.01
MP × O × TI	2,292	3.68	0.026	0.02	3.44	0.033*	0.02
MP × L × TI	2,292	0.11	0.895	< 0.01	2.95	0.054	0.02
O × L × TI	2,292	2.89	0.061	0.02	4.09	0.018*	0.03
MP × O × L × TI	2,292	0.99	0.369	0.01	0.58	0.559	< 0.01

MP = manipulation phase, O = orientation, L = line length, TI = target interval.

Asterisks indicate qualitatively different results from the main analyses.

## Footnotes

1 The qualitative pattern of results was similar when no trials were excluded. However, even more participants (*n* = 40) should have been excluded from analyses in this case due to non-significant or negative regression slopes.

2 As in Experiment 1, a 3 *SD* threshold would have resulted in an even higher number of excluded participants (*n* = 72).

## References

[CR1] Alards-Tomalin D, Leboe-McGowan JP, Shaw JD, Leboe-McGowan LC (2014). The effects of numerical magnitude, size, and color saturation on perceived interval duration. Journal of Experimental Psychology: Learning, Memory, and Cognition.

[CR2] Avery GC, Day RH (1969). Basis of the horizontal-vertical illusion. Journal of Experimental Psychology.

[CR3] Birngruber T, Ulrich R (2019). Perceived duration increases not only with physical, but also with implicit size. Journal of Experimental Psychology: Learning Memory and Cognition.

[CR4] Boroditsky L (2000). Metaphoric structuring: Understanding time through spatial metaphors. Cognition.

[CR5] Bratzke D, Ulrich R (2019). Temporal reproduction within and across senses: Testing the supramodal property of the pacemaker-counter model. Journal of Experimental Psychology: Human Perception and Performance.

[CR6] Bratzke D, Birngruber T, Durst M, Schröter H (2017). Filled and empty motor reproductions of filled and empty intervals: Is there also a filled-reproduction illusion?. Attention, Perception, & Psychophysics.

[CR7] Brown SW (1995). Time, change, and motion: The effects of stimulus movement on temporal perception. Perception & Psychophysics.

[CR8] Cai ZG, Connell L (2015). Space-time interdependence: Evidence against asymmetric mapping between time and space. Cognition.

[CR9] Cai ZG, Connell L (2016). On magnitudes in memory: An internal clock account of space–time interaction. Acta Psychologica.

[CR10] Cai ZG, Wang R (2014). Numerical magnitude affects temporal memories but not time encoding. PLoS ONE.

[CR11] Cai, Z. G., & Wang, R. (2022). Cross-dimensional magnitude interaction is modulated by representational noise: evidence from space–time interaction. *Psychological Research,**86*(1), 196–208. 10.1007/s00426-020-01472-4 33580821

[CR12] Cai ZG, Wang R, Shen M, Speekenbrink M (2018). Cross-dimensional magnitude interactions arise from memory interference. Cognitive Psychology.

[CR13] Casasanto D, Boroditsky L (2008). Time in the mind: Using space to think about time. Cognition.

[CR14] Chang AYC, Tzeng OJ, Hung DL, Wu DH (2011). Big time is not always long: Numerical magnitude automatically affects time reproduction. Psychological Science.

[CR15] Coren S, Girgus JS, Erlichman H, Hakstian AR (1976). An empirical taxonomy of visual illusions. Perception & Psychophysics.

[CR16] Creelman CD (1962). Human discrimination of auditory stimuli. Journal of the Acoustical Society.

[CR17] Eikmeier V, Schröter H, Maienborn C, Alex-Ruf S, Ulrich R (2013). Dimensional overlap between time and space. Psychonomic Bulletin & Review.

[CR18] Faul, F., Erdfelder, E., Lang, A.-G., & Buchner, A. (2007). G*Power 3: A flexible statistical power analysis program for the social, behavioral, and biomedical sciences.* Behavior Research Methods, 39*, 175–191. 10.3758/bf03193146 17695343

[CR19] Gibbon J, Church RM, Meck WH, Gibbon J, Allan L, Sninsky JJ, White TJ (1984). Scalar timing in memory. Timing and time perception.

[CR20] Haspelmath, M. (1997). *From space to time: Temporal adverbials in the worlds languages*. Lincom Europa.

[CR21] Künnapas TM (1955). An analysis of the "vertical-horizontal illusion". Journal of Experimental Psychology.

[CR22] Matthews, W. J., & Meck, W. H. (2016). Temporal cognition: Connecting subjective time to perception, attention, and memory. *Psychological Bulletin,**142*, 865–907. 10.1037/bul0000045 27196725

[CR23] Mo SS, Michalski VA (1972). Judgment of temporal duration of area as a function of stimulus configuration. Psychonomic Science.

[CR24] Morey, R. D. (2008). Confidence intervals from normalized data: A correction to cousineau (2005). *Tutorial in Quantitative Methods for Psychology,**4*(2), 61–64. 10.20982/tqmp.04.2.p061

[CR25] Morys-Carter, W. L. (2021, May 18). *ScreenScale* [Computer software]. Pavlovia. 10.17605/OSF.IO/8FHQK

[CR26] Oliveri, M., Vicario, C. M., Salerno, S., Koch, G., Turriziani, P., Mangano, R., ... & Caltagirone, C. (2008). Perceiving numbers alters time perception. *Neuroscience Letters*, *438*(3), 308–311. 10.1016/j.neulet.2008.04.051 18486340

[CR27] Ono F, Kawahara JI (2007). The subjective size of visual stimuli affects the perceived duration of their presentation. Attention, Perception & Psychophysics.

[CR28] Peirce JW, Gray JR, Simpson S, MacAskill MR, Höchenberger R, Sogo H, Kastman E, Lindeløv J (2019). PsychoPy2: Experiments in behavior made easy. Behavior Research Methods.

[CR29] Rammsayer, T. H., & Verner, M. (2014). The effect of nontemporal stimulus size on perceived duration as assessed by the method of reproduction. *Journal of Vision,**14*(5), 17. 10.1167/14.5.17 10.1167/14.5.17 24879864

[CR30] Rammsayer, T. H., & Verner, M. (2015). Larger visual stimuli are perceived to last longer from time to time: The internal clock is not affected by nontemporal visual stimulus size. *Journal of Vision,**15*(3), 5. 10.1167/15.3.5 10.1167/15.3.5 25758710

[CR31] Rammsayer, T. H., & Verner, M. (2016). Evidence for different processes involved in the effects of nontemportal stimulus size and numerical digit value on duration judgments. *Journal of Vision,**16*(7), 13. 10.1167/16.7.13 10.1167/16.7.13 PMC4900137 27191941

[CR32] Starr A, Brannon EM (2016). Visuospatial working memory influences the interaction between space and time. Psychonomic Bulletin & Review.

[CR33] Thomas EA, Cantor NE (1975). On the duality of simultaneous time and size perception. Perception & Psychophysics.

[CR34] Togoli, I., Bueti, D., & Fornaciai, M. (2022). The nature of magnitude integration: Contextual interference versus active magnitude binding. *Journal of Vision,**22*(11), 11. 10.1167/jov.22.11.11 10.1167/jov.22.11.11 PMC9587468 36259675

[CR35] Treisman M (1963). Temporal discrimination and the indifference interval: Implications for a model of the" internal clock". Psychological Monographs.

[CR36] Ulrich R, Eikmeier V, de la Vega I, Ruiz Fernández S, Alex-Ruf S, Maienborn C (2012). With the past behind and the future ahead: Back-to-front representation of past and future sentences. Memory & Cognition.

[CR37] Vicario CM (2011). Perceiving numbers affects the subjective temporal midpoint. Perception.

[CR38] Walsh V (2003). A theory of magnitude: Common cortical metrics of time, space and quantity. Trends in Cognitive Sciences.

[CR39] Wearden JH (2016). The psychology of time perception.

[CR40] Winter B, Marghetis T, Matlock T (2015). Of magnitudes and metaphors: Explaining cognitive interactions between space, time, and number. Cortex.

[CR41] Xuan, B., Zhang, D., He, S., & Chen, X. (2007). Larger stimuli are judged to last longer. *Journal of Vision,**7*(10), 2. 10.1167/7.10.2 10.1167/7.10.2 17997671

[CR42] Yates, M. J., Loetscher, T., & Nicholls, M. E. R. (2012). A generalized magnitude system for space, time, and quantity? A cautionary note. *Journal of Vision,**12*(7), 9. 10.1167/12.7.9 10.1167/12.7.9 22822089

[CR43] Zakay D, Block RA (1997). Temporal cognition. Current Directions in Psychological Science.

